# Dynamics of 2013 Sudden Stratospheric Warming event and its impact on cold weather over Eurasia: Role of planetary wave reflection

**DOI:** 10.1038/srep24174

**Published:** 2016-04-07

**Authors:** Debashis Nath, Wen Chen, Cai Zelin, Alexander Ivanovich Pogoreltsev, Ke Wei

**Affiliations:** 1 Center for Monsoon System Research, Institute of Atmospheric Physics, Chinese Academy of Sciences, Beijing 100190, China; 2 Russian State Hydrometeorological University, Maloohtingsky 98, St. Petersburg, 195196, Russia

## Abstract

In the present study, we investigate the impact of stratospheric planetary wave reflection on tropospheric weather over Central Eurasia during the 2013 Sudden Stratospheric Warming (SSW) event. We analyze EP fluxes and Plumb wave activity fluxes to study the two and three dimensional aspects of wave propagation, respectively. The 2013 SSW event is excited by the combined influence of wavenumber 1 (WN1) and wavenumber 2 (WN2) planetary waves, which makes the event an unusual one and seems to have significant impact on tropospheric weather regime. We observe an extraordinary development of a ridge over the Siberian Tundra and the North Pacific during first development stage (last week of December 2012) and later from the North Atlantic in the second development stage (first week of January 2013), and these waves appear to be responsible for the excitation of the WN2 pattern during the SSW. The wave packets propagated upward and were then reflected back down to central Eurasia due to strong negative wind shear in the upper stratospheric polar jet, caused by the SSW event. Waves that propagated downward led to the formation of a deep trough over Eurasia and brought extreme cold weather over Kazakhstan, the Southern part of Russia and the Northwestern part of China during mid-January 2013.

In the recent decades, several studies have discussed the impact of stratospheric dynamic processes on tropospheric climate variability, in the light of downward coupling between the stratosphere and troposphere^1^,^2^,^3^. The studies mainly demonstrated the downward propagation of zonal mean zonal wind anomalies^4^,^5^,^6^ and stratospheric influences due to strong wave-mean flow interaction^1^,^2^,^7^,^8^. In particular, there are evidences of downward propagation of stratospheric anomalies in the strength of the polar vortex to the lower stratosphere^7^,^9^, which have significant impact on the tropospheric weather regimes.

The statistical works by Perlwitz and Harnik^10^ relates the downward propagation of stratospheric anomalies to the reflection of planetary waves from the stratosphere^10^. However, the theory of planetary wave reflection was first proposed by Hines^11^ and Geller and Alpert^12^. Since then the subject of reflection and its impact on the tropospheric weather has become more popular in the recent decades^1^,^2^,^13^,^14^,^15^. Holton and Mass^16^ and Kodera *et al*.^17^ discussed the eastward shift in the region of strongest interaction between the upward propagating Rossby waves and stratospheric zonal flow^16^,^18^. Kodera *et al*.^17^ showed that, during the SSW wave packets propagating upward were reflected back by the negative wind shear in the upper stratospheric westerly jet. This led to a poleward and downward progression of the mean flow perturbations^19^ in the American-Atlantic sector. Due to gradual increase in the air density, downward, the impact of the reflected component of planetary waves was long considered to be weak on the troposphere. But the recent studies have found its significance in the tropospheric regime and discussed the downward coupling processes of WN1 and WN2 in the light of stratospheric dynamical states^10^,^19^,^20^.

According to Nakagawa and Yamazaki^21^, the SSW events that propagate into the troposphere are associated with stronger and upward propagating WN2 and WN1 EP fluxes. On the other hand, the events which do not propagate down to the troposphere are associated with weakened WN2 and enhanced WN1 upward EP fluxes. Additionally, they^21^ suggest that the warming types can tell whether the downward propagation occurs or not. Pure WN1 and WN2 warming represents displacement and splitting of the vortex, respectively. Subjective inspection of each SSW event at 50 hPa reveals that 14 and 37 of the cases are splitting and displacement events, respectively. Among the 14 splitting events, 11 are followed by tropospheric polar warming and 3 by cooling. While among the 37 displacement events, 17 are followed by tropospheric polar warming and 20 by cooling^21^. In a separate study, Mitchell *et al*.^22^ finds a strong link between stratospheric variability and anomalous weather patterns at the earth’s surface. Specifically, during the weak vortex event, anomalies can descend from the upper stratosphere to the surface on time scales of weeks. Subsequently the outbreak of cold-air events has been noted in high northern latitudes. Moreover, they found that the vortex splitting events are correlated with surface weather and lead to positive (1.5 K) and negative (−3 K) temperature anomalies over eastern North America and over Eurasia, respectively. Furthermore, in a recent study Kodera *et al*.^23^ show that differences in the recovery phase can be used to classify the SSW events into two types. The first is the absorbing type of SSW, with longer timescale and larger meridional extent due to the persistent incoming planetary waves from the troposphere. The other is the reflecting type, which is characterized by a quick termination of the warming episode due to the reflection of planetary waves in the stratosphere. This eventually leads to an amplification of tropospheric planetary waves.

Using the lagged correlation analysis, Perlwitz and Harnik^19^ statistically categorized the stratospheric winter states into reflective and non-reflective states. They have defined the reflective index based on the zonal mean zonal wind difference between 2 hPa and 10 hPa, averaged over 58^o^–74^o^N and over time (January-March mean of zonal mean field). The index values are highly sensitive to the latitude boundaries chosen, avoiding the easterly winds in the subtropics and the high latitudes. This is because, reflection forms when the stratospheric polar night jet is peaking up in the mid stratosphere. It physically signifies the average vertical wind shear in the high latitude stratosphere. In one state, wave activity is reflected back down to the troposphere and affects the structure of the tropospheric planetary waves. While in the other state, wave activity gets deposited in the stratosphere, resulting in the strong wave-mean flow interaction. During the reflective background state, Perlwitz and Harnik^19^ had found the dominance of WN1 and zonal mean component over WN2 component, but in the shorter and longer time scale, respectively. Additionally, they^19^ also suggest that the dominant process is upward propagation of WN1, with some contribution of WN2 in the shorter timescale. This relative dominance of WN1 reflection can be understood from the model results by Harnik and Lindzen^24^. The meridional wavenumber, both for WN1 and WN2 is similar and is oriented vertically with an equatorward tilt along the jet axis. But the index of refraction for vertical wave propagation (*n*^*2*^_*ref*_) exhibits larger values for WN1 compared to WN2, result in larger vertical wavenumber values (larger propagation region). The horizontal reflecting surface is at much lower altitude for WN2 and therefore WN2 has much smaller amplitude i.e. the geopotential height peaks roughly at the height of the reflecting surface. Moreover, the WN1 has turning surfaces at six and seven scale heights, resulting in a region of evanescence there. Since this region is much smaller than one vertical wavelength, the WN1 tunnels through it easily^24^.

Furthermore, Perlwitz and Harnik^10^ observed a westward and eastward phase tilt of the WN1 regression patterns (leading mode) with increasing altitude for negative and positive time lags, respectively; and the eastward tilting feature of the wave axes are consistent with the downward reflection of the planetary waves. Recent study by Shaw *et al*.^14^ showed that the downward coupling of WN1 occurs in the presence of both a vertical reflecting surface in mid-upper stratosphere and high latitude meridional waveguide in the lower stratosphere. Additionally, Shaw *et al*.^14^ highlights the distinction between wave reflection and downward wave coupling between the stratosphere and troposphere. Nevertheless, from these analyses it is difficult to quantify the changes in upward wave propagation in the stratosphere and reflection to the troposphere. This is because; it is highly challenging to separate the impact of reflected waves from the tropospheric variation itself.

In most of the previous studies^10^,^19^, waves are defined as departure from a time mean field which in general includes the upward propagating planetary waves. Therefore, the downward wave flux anomaly does not necessarily means downward wave propagation but a less upward propagation. Furthermore, the decomposition of waves into a single wavenumber makes it difficult to distinguish the reflected wave component from that generated in the troposphere^17^. To resolve this issue, Kodera *et al*.^17^ had defined the waves as departure from the zonal mean field (eddy fluxes) and the downward wave fluxes really means downward propagation of the planetary waves. Therefore, in the present analysis, we have considered the eddy fluxes i.e. the total wavenumber to provide clear evidences of the reflected waves from the stratosphere.

Coughlin and Tung^25^ and Kodera *et al*.^17^ observed the reflection of planetary waves in conjunction with major SSW and have discussed the impact on the tropospheric weather regimes. In some cases, during a SSW event (particularly induced by WN1), the upper stratospheric zonal mean zonal wind is weakened substantially, while the lower stratospheric jet remains strong. This creates a negative vertical wind shear in the upper stratosphere and provides a favorable background for the reflection of upward propagating planetary waves. Coughlin and Tung^25^ reported the changes in WN1 field in the troposphere due to the reflection of planetary waves from the stratosphere. While Kodera *et al*.^17^ considered the total wavenumber field to demonstrate the reflection of planetary waves during the final warming of March 2007, in which upward propagating waves from Eurasia were reflected back to the North American sector, resulting in very cold weather over the Northeast coast of the North American continent^17^. Recently, Harada *et al*.^26^ has addressed the upward propagation of the wave packets from Alaska and Siberia during 2009 sudden stratospheric warming event. Although, several mechanisms are proposed, the transmission of eddies to the troposphere during the winter months are still not well understood.

In the present study, we explore vertical wave propagation and coupling during the 2013 SSW, an event excited by the combined influence of WN1 and later WN2 in the prewarming stage of the SSW. Charlton and Polvani^27^ had distinguished between different types of SSWs, based on the synoptic structure in the middle stratosphere. One is vortex displacement type and the other is vortex split type. They^27^ found that about 46% of the warming events lead to a splitting of the stratospheric polar vortex. Moreover, in the vortex displacement type, anomalous wave activity begins 20 days prior to the stratospheric wind reversal and the heat flux during the warming event is primarily due to the WN1. On the other hand, in the vortex splitting type, a precursor heat flux anomaly 30 days prior to the central date (due to WN1) is followed by a second heat flux anomaly 10 days before the central date (due to WN2) is predominant. In a separate study by Harada *et al*.^26^ had identified (based on WMO criteria) 22 SSW events between 1978 and 2009, and found that about 68% of the cases are due to WN1. On the other hand WN2, WN1 + WN2 and WN1 + WN3 account for 18%, 9% and 4.5% of the cases, respectively. This makes the 2013 event an uncommon one (1985/86, 1987/88 and 2012/13) and seems to have deep impact on the tropospheric cold weather over the central part of the Eurasian continent.

During the SSW 2013, it has found that, in the initial stage (last week of December 2012) upward fluxes from the Siberian Tundra and North Pacific region and later from the North Atlantic region (first week of January 2013) is responsible for the excitation of WN2 pattern. The wave packets are then reflected downward toward the central part of the Eurasian continent due to strong negative wind shear in the upper stratospheric polar jet. The result was extreme cold weather over central Eurasia during mid-January 2013. Next section describes the data and methodologies used here. The main results are thoroughly discussed in following section and the conclusions are summarized in final section.

## Data and Methodology

### Data

This study uses daily reanalysis horizontal wind, temperature and geopotential data from the European Centre for Medium-Range Weather Forecasts (ECMWF) Re-Analysis Interim (ERA-Interim)^28^,^29^ from December 2012 and January 2013. The data is available at 37 pressure levels from 1000 hPa to 1 hPa with horizontal resolution of 1.5^o^ × 1.5^o^.

### Methodology

#### The Reflective Index (RI)

Perlwitz and Harnik categorized the reflective and nonreflective basic states for planetary wave reflection based on the zonal mean zonal wind difference between 2 hPa and 10 hPa, averaged over 58^o^–74^o^N and over time (January-March)^19^. They had defined the reflective index as


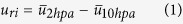


Positive and negative index corresponds to the nonreflective and reflective basic states, respectively. Here we have calculated the daily reflective index to understand the temporal variability of the wave reflection in the mid-stratosphere.

#### Least square fitting analysis (LSF)

To extract the WN1 and WN2 components from the eddy geopotential perturbations we have applied the LSF technique for spectral analysis^30^. This method is used to fit a set of observations, *y*_*i*_ at times *i* = 1, 2 …N, to the equation given by


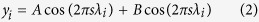


Where, *s* is the wavenumber, *λ*_*i*_ are the longitudes and *A* and *B* are the coefficients to be fitted. The individual wave components are then computed using the empirical relation,


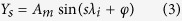


Where, *A*_*m*_ is the amplitude and φ is the phase, estimated by means of LSF analysis.

## Results

### Stratospheric sudden warming of 2013

**Figure 1 f1:**
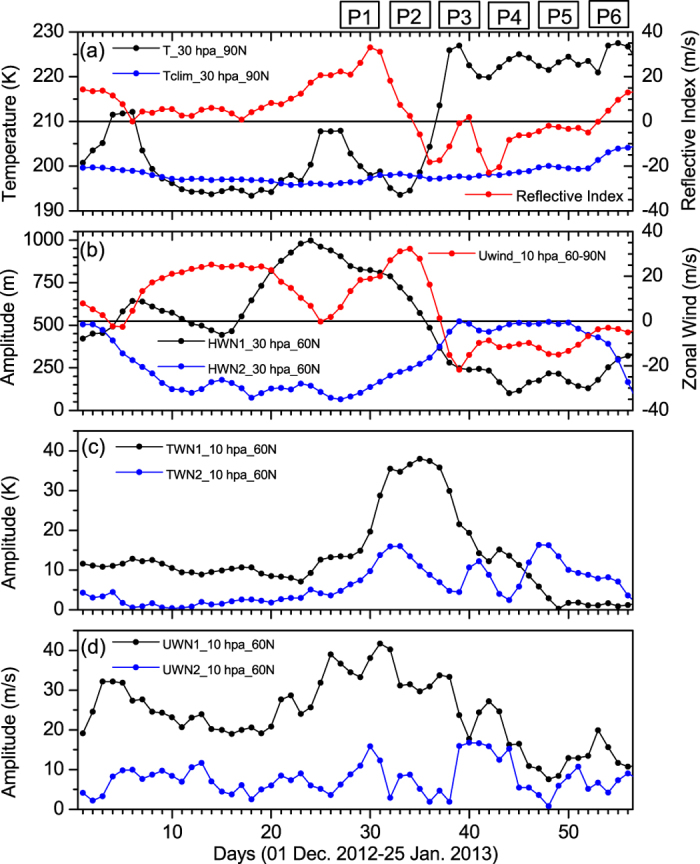
Temporal evolution of SSW event, reflective index and planetary WN1 and WN2 amplitudes. (**a**) Timeseries of temperature at 30 hPa & 90^o^N (black line, left panel), climatological mean temperature at 30 hPa & 90^o^N (blue line, left panel), and reflective index (red line, right panel), (**b**) time-series of height WN1 (black line) and height WN2 (blue line) at 30 hPa and 60^o^ N. The right panel represents zonal mean zonal wind at 10 hPa (red line, mean over 60^o^–90^o^ N). (**c**) time-series of temperature WN1 (black line) and temperature WN2 (blue line) at 10 hPa and 60^o^ N, (**d**) represents same as (**c**) but for zonal wind. P1-P6 represents the pentads. The subplots are generated using **ORIGIN** software (Version 7, URL: http://www.originlab.com/).

In our analysis, ERA interim temperature data are used to obtain the state of the high-latitude winter hemisphere. In general, SSW is defined as the deviation of the north polar temperature at 30 hPa from its long term climatological mean value. To illustrate the changes, temporal variation of the north polar temperature at 30 hPa during December 2012-January 2013 is shown in Fig. 1a (black line). The daily climatological mean temperature at 90^o^N is overlapped on the temporal trend in temperature at 30 hPa (blue line, Fig. 1a). A sudden increase in polar temperature from 195 K on 2^nd^ January to 230 K on 8^th^ January, nearly 30 K higher than the climatological mean temperature has been noticed and remained warmer than average for 3 weeks. In the right panel of Fig. 1a, we have plotted the daily march of the reflective index (red line). It clearly reflects the stratospheric state as reflective (negative) from 4^th^ January onwards and persists until 22^nd^ January, with a maximum around 11^th^, when the polar vortex breaks down completely (not shown here). From 23^rd^ January onwards the night jet seems to stabilize and the index becomes positive. Therefore, in the first week of January 2013 i.e. in the wake of a major warming event the stratosphere provides a favorable background for the planetary waves to reflect back to the troposphere.

Figure 1b depicts the temporal behavior of 30 hPa WN1 (black line) and WN2 (blue line) amplitude at 60^o^N for the same period mentioned above. This 2013 SSW event, more or less follows the ‘type A’ warming pattern i.e. a small WN2 pulse is followed by a large peak in WN1, and finally another WN2 pulse of variable strength at about the time of the main temperature rise^31^. December was characterized by large WN1 pulse, but in the first week of January WN2 become dominant at the same time of the main temperature rise (Fig. 1a,b). This strong WN1 pulse acts as precondition for the WN2 to propagate upward into the stratosphere. Daily march of zonal mean zonal wind at 10 hPa and mean over 60^o^–90^o^N (red line) is overlapped on the temporal trend in wave amplitudes. In the prewarming stage, high latitude zonal wind is westerly and WN1 is predominant in the stratosphere. During the peak warming stage, the polar night jet reverses completely to easterly and WN2 is dominant in the high latitude stratosphere (Fig. 1b). This is indicative to the fact, that in the prewarming stage WN2 is trapped in the troposphere and only WN1 propagate upward to the stratosphere. But when the polar jet weakens, WN2 propagate upward and the actual warming begins in the high latitude stratosphere. Furthermore, we have plotted the temperature and zonal wind WN1 (black line) and WN2 (blue line) at 10 hPa in Fig. 1c[Fig f1]d, respectively. Both for temperature and zonal wind WN1 amplitude is much higher than WN2 amplitude and it maximizes just prior to the peak warming event.

Based on the time evolution of the major SSW we have fragmented the entire event from 27^th^ December to 25^th^ January into 6 pentads (P1–P6), to carefully analyze the prewarming and the warming phase, separately. The periods are as follows viz. 27 Dec.–31 Dec. 2012 (P1), 01 Jan.–05 Jan. 2013 (P2), 06 Jan.–10 Jan. 2013 (P3), 11 Jan.–15 Jan. 2013 (P4) and 16 Jan.–20 Jan. 2013 (P5).

### Two dimensional propagation of Eddy flux

**Figure 2 f2:**
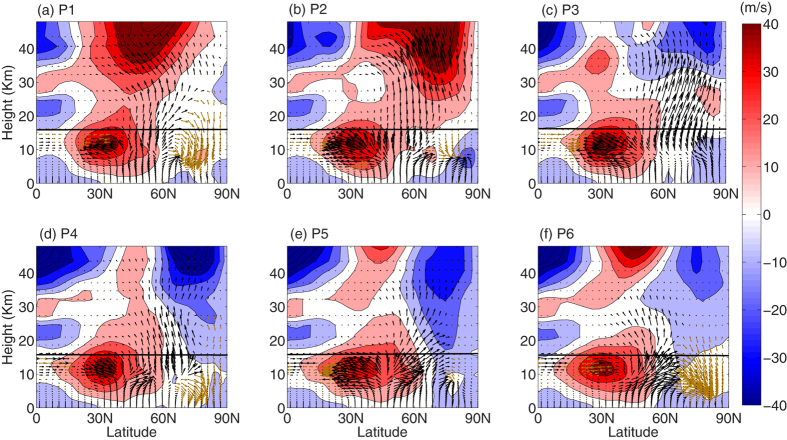
Pentad mean EP flux vector (eddy component). Latitude-Height section of EP flux vectors (eddy component), the black and brown arrows indicate the upward and the downward propagating vectors, respectively. The background contour represents the zonal mean zonal wind (m/s). Subplots (**a–f**) represent the pentads, from P1 to P6. The black lines in each of the subplots indicate the 100 hPa level. The subplots in the figure are generated using the **MATLAB** software (version: R2012b (8.0.0.783) & URL: www.mathworks.com/products/matlab/?s_tid=srchtitle).

Figure 2 depicts the latitude-height section of pentad mean EP flux vectors (stationary waves) overplotted on zonal mean zonal wind. The upward and downward propagation of the fluxes are marked with black and brown arrows, respectively. The EP flux vectors^32^ are parallel to the local group velocity and its convergence indicates the retardation of the zonal flow due to wave forcing^17^. However, EP-flux vectors do not show propagation exactly; rather they indicate potential propagation in an idealized sense. During P1 and P2 (Fig. 2a[Fig f2]b), planetary waves propagate upward from the troposphere between 40^o^–60^o^N and diverge poleward and equatorward above the tropopause such that they travel both into the polar stratosphere and the tropical upper troposphere and lower stratosphere (UTLS). These features are consistent with that of Huang and Gambo^33^. For P1, the westerly wind appears to be strong over midlatitude, which gradually shifts over the high latitudes during P2 and thereby provides a favorable background for the waves to propagate upward^34^ and focus over the polar cap. Strong upward propagation of the planetary waves during P2, leads to the weakening of the upper stratospheric polar night jet. During P3 (Fig. 2c), the upper stratospheric zonal wind north of 60^o^N reverses to easterly and attains its maximum value during P4 (Fig. 2d). Subsequently, during P4 planetary waves seems to overturn in the high latitude mid stratosphere and it continues until P6. Along with the upward propagation of the WN2 component, during P3 to P5, these background conditions in the high latitude stratosphere are explicitly suitable for the reflection of planetary waves down to the troposphere. During P6, the high latitude stratosphere is devoid of upward propagation and is dominated by the reflected components.

### Wave propagation in the three dimensional plane

**Figure 3:**
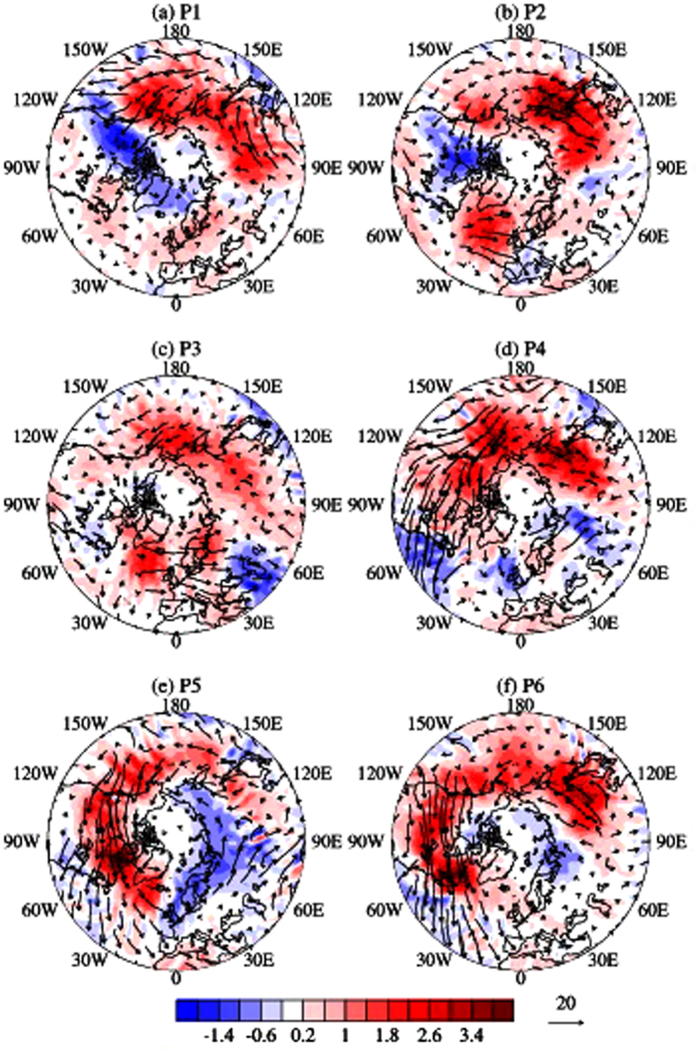
Pentad mean 3 dimensional Plumb wave activity fluxes (eddy component) at 100 hPa. Longitude-Latitude section of vertical (color contour) and horizontal (arrows) component of Plumb wave activity fluxes at 100 hPa from P1 (**a**)–P6 (**f**). The red and blue colors indicate the upward and downward fluxes, respectively. All the fluxes represent the eddy components. The maps in the figure are generated using the **MATLAB** software (version: R2012b (8.0.0.783) & URL: www.mathworks.com/products/matlab/?s_tid=srchtitle).

The above is the conventional view of the two-dimensional propagation of planetary waves, but in the real atmosphere the waves can propagate zonally also^35^. Hence, the zonal mean fluxes do not provide the complete information of planetary wave propagation, particularly during SSW events, which include cumulative effects of rather localized propagation of wave packets from the troposphere. To investigate the zonal evolution of the wave packets forced by the topography, three dimensional stationary wave activity fluxes are analyzed for all the pentads mentioned before. Figure 3 shows the horizontal distribution of the vertical (color contour) and horizontal component (arrows) of the Plumb wave activity fluxes at 100 hPa. Since we are interested to explore wave reflection and its impact on troposphere, we restrict our further discussion to P1–P6 only; the period until which the background stratospheric state is reflective.

#### Horizontal distribution of Plumb wave activity fluxes (100 hpa)

From Fig. 3, it can be seen that the upward and downward propagation occurs in different geographical locations. During P1 and P2, i.e., in the development phase of SSW, the polar night jet is westerly and the background strongly favors upward propagation of planetary waves from the troposphere. On P1, upward propagation can be seen from the Siberia and the North Pacific (Fig. 3a), whereas on P2, another bunch of wave packets are propagating upward from the North Atlantic (Fig. 3b). The first one contribute to the eastward and poleward wave propagation, whereas, the later one to the equatorward propagation. These features are consistent with the poleward and equatorward propagation of planetary waves seen diverging out of the midlatitude tropopause region in Fig. 2.

During P3–P4 (Fig. 3c,d) i.e., in the warming phase with reflective stratospheric background, the zonal wind reverses completely from westerly to easterly and the planetary waves propagate eastward towards the continents from the oceans. During P4, when the easterly jet is strongest in the high latitude upper stratosphere the Siberian center reactivates and wave packets start propagating eastward and poleward with evidence of reflection (Fig. 2d) over central Eurasia (60°–90°E). During P5, the downward and poleward (Fig. 2e) component of the planetary waves appears to be stronger over the wider region of the Eurasian continent (Fig. 3e). It is worth mentioning in this context, that during P4 and P5 planetary waves seen diverging equatorward and downward between 60° and 90°E. However, the EP fluxes in Fig. 2d,e are mainly dominated by downward component of the poleward fluxes, which means the zonal mean component might have masked the regional features. For P6, due to weakening of the easterly wind (Fig. 2f), the background tends to be non-reflective and the downward component of the planetary waves weakens subsequently (Fig. 3f) with gradual propagation towards east.

#### Longitude-Height section of Plumb wave activity fluxes (50^o^N to 80^o^N)

**Figure 4:**
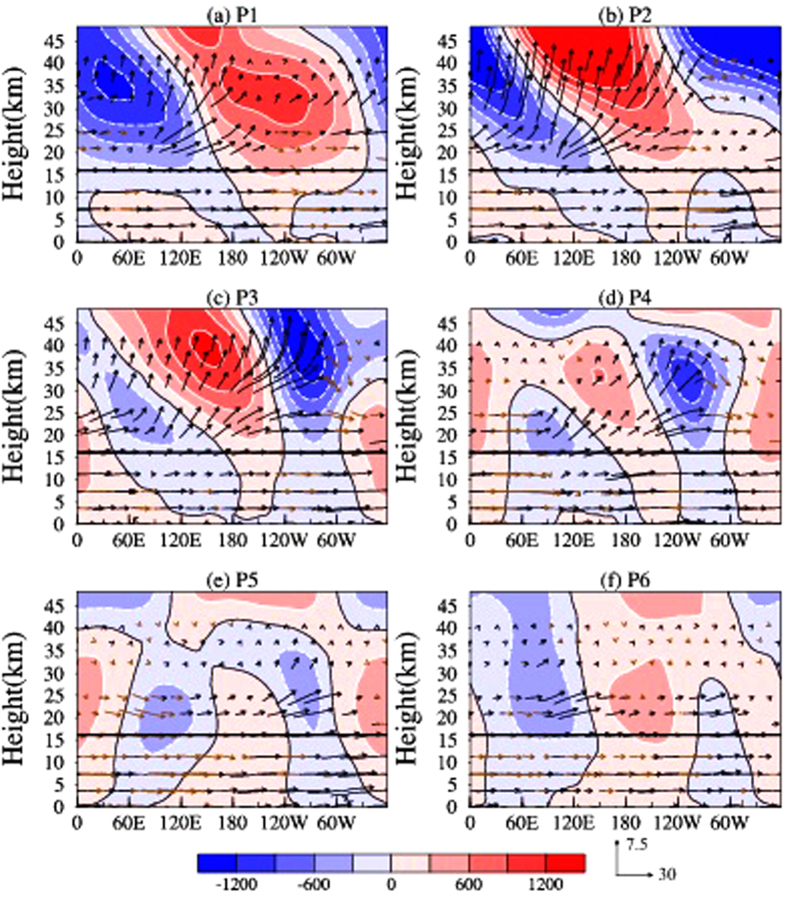
Pentad mean Plumb wave activity fluxes (eddy component) averaged between 50^o^–80^o^N. Longitude-Height section of Plumb wave activity fluxes (arrows) and geopotential heights (color contour) averaged between 50^o^–80^o^N and from P1 (**a**) to P6 (**f**). All the fluxes represent the eddy components. The black and brown arrows indicate the upward and downward fluxes, respectively. The subplots in the figure are generated using the **MATLAB** software (version: R2012b (8.0.0.783) & URL: www.mathworks.com/products/matlab/?s_tid=srchtitle).

Since the upward and downward components are concentrated mostly between 50^o^N to 80^o^N, we plot the longitude-height section of Plumb wave activity flux (arrows) and eddy geopotential height (color shading) in Fig. 4, averaged over the latitude band mentioned above. For P1, Fig. 4a depicts the vertical propagation of planetary waves, but only the WN1 component seems to propagate up into the stratosphere along with the westerly jet and the WN2 component is trapped in the troposphere. This can be seen as one and two troughs pattern in eddy geopotential height fields in the stratosphere and troposphere, respectively. During P1, the lower stratospheric trough over Siberian Tundra is located around 120^o^E, tilts westward with increasing altitudes to 30^o^E at 10 hPa. This westward tilt in the trough line during P1 indicates upward propagation of the Rossby waves and is seen in the vertical component of the Plumb flux (Fig. 3a). For P2, when the westerly jet weakens and centers over the polar cap (see Fig. 2b), the background seems favorable for waves to propagate up into the stratosphere from the North Atlantic.

For P3, when the upper stratospheric wind reverses to easterly, WN2 propagates upward (Fig. 4c).This can clearly be seen from the eastward tilt and two trough pattern in the eddy geopotential height, particularly at 60^o^ W. During P4, the upper stratospheric easterly wind is strongest in the high latitudes and planetary wave seems propagating downward and poleward (Fig. 2d), east of 60^o^W (Fig. 4d). Interestingly, the trough (below 30 km) around 100^o^E exhibits a complex pattern, with eastward and westward tilt around 50^o^–100^o^E and 100^o^–180^o^E, respectively (Fig. 4d). Subsequently, during P4 planetary wave seems reflected back to the troposphere between 50^o^–120^o^E and from 34 km. During P5, the trough around 100^o^E tilts completely eastward and WN2 seems propagating further eastward and downward (~850 hPa) to the troposphere (Fig. 4e). During P6, the downward propagation of the planetary wave weakens significantly and the stratosphere (30–48 km) is dominated by WN1 component (Fig. 4f).

**Figure 5:**
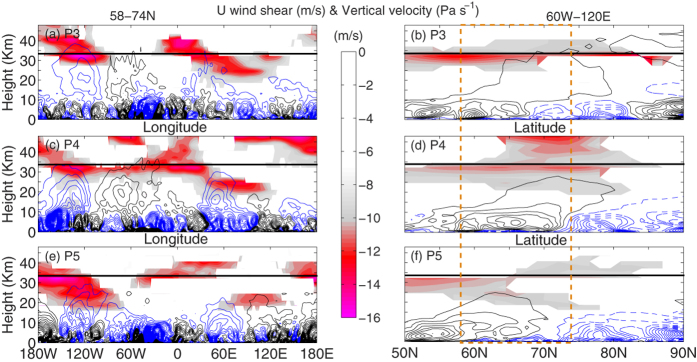
Negative wind shear and vertical velocity. (**a,c,e**) represent the longitude-height section (average over 58^o^–74^o^N) of negative wind shear (color shading) and vertical velocity (line contours) during P3, P4 and P5, respectively. The black solid and blue dashed contours represent the upward and downward component of vertical velocity. (**b**,**d**,**f**) represent the same but the latitude-height section (average over 60^o^ W–120^o^E). The black line at each subplot indicates 34 km (~5 hPa). The latitudinal band between 58^o^ and 74^o^N is marked with yellow and dashed box in (**b**,**d**,**f**). The subplots in the figure are generated using the **MATLAB** software (version: R2012b (8.0.0.783) & URL: www.mathworks.com/products/matlab/?s_tid=srchtitle).

In order to illustrate the physical mechanism of planetary wave reflection, the role of negative wind shear during P3, P4 and P5 is explained in Fig. 5. Perlwitz and Harnik^10^ shows that when the polar night jet peaks up in the high-latitude mid-stratosphere planetary wave reflects back to the troposphere. The existence of reflecting surfaces and a strong downward signal in the troposphere is a result of two coincident features- the formation of a reflecting surface for vertical propagation around 5 hPa and a meridional waveguide in the middle and lower stratosphere. This meridional waveguide channels the reflected waves to the high latitude troposphere, which prevents dispersion of the waves in the meridional direction before they reach the mid-troposphere^10^. The vertical reflection surface and meridional waveguide forms as a result of increased vertical and meridional curvature of vertical wind along the jet axis, respectively.

To establish the hypothesis the longitude-height section of vertical shear (negative) of zonal wind (mean over 58^o^–74^o^N) for P3, P4 and P5 are shown in Fig. 5a,c,e, respectively. To observe the vertical wind curvature we overplotted the longitude-height section of vertical wind over the same latitude band mentioned above. During P3, when the polar jet reverses to easterly the regions of maximum negative wind shear (<−10 m/s) tilts eastward from 90^o^W at 30 km to 180^o^W at 48 km and from 120^o^E at 20 km to 0^o^ at 45 km (Fig. 5a). Subsequently, increased upward component of vertical velocity at 60^o^W is consistent with upward propagation of WN2 around 60^o^W (Fig. 4c). During P4, when the easterly wind is strongest in the polar upper stratosphere, a strong negative wind shear surface around 34 km (180^o^W–60^o^E) is consistent with the formation of a reflecting surface at 5 hPa by Perlwitz and Harnik. Moreover, during P4 the negative wind shear is stronger in the upper stratospheric heights (60^o^W–60^o^E & above 40 km) and it extends downward from 60^o^E and 34 km to 120^o^E and 20 km. As a result, an increased eastward curvature in the upward component of vertical wind at 60^o^W (below 34 km) and downward component between 60^o^E and 120^o^E is in agreement with the eastward propagation of upward planetary waves and downward propagation of reflected planetary waves, respectively (Fig. 4d). During P5, gradual weakening of the easterly jet (Fig. 4e) results in weak negative wind shear in the stratosphere (Fig. 5e). Subsequently, the upward component of the vertical velocity at 60^o^W disappears and the downward component remains stronger, particularly around 60^o^E and in the troposphere (below 20 km).

According to Perlwitz and Harnik^10^, for planetary waves to reflect, formation of a meridional waveguide is consistent with an increased meridional curvature in vertical velocity. Therefore, we have plotted the latitude-height section of negative wind shear and vertical velocity (mean over 60^o^W–120^o^E) for P3, P4 and P5 in Fig. 5b,d,f, respectively. During P3, a region of high negative wind shear can be seen around 34 km and the vertical velocity exhibits an upward and poleward curvature (Fig. 5b), which is consistent with the upward propagating planetary waves, north of 60^o^N (Fig. 2c). During P4, the easterly jet is strongest in the polar upper stratosphere (Fig. 2d), which is consistent with strong negative wind shear in the mid and upper stratosphere (25–48 km, 65^o^–80^o^N). Additionally, the poleward curvature in negative wind shear from 32 km and 65^o^N to 20 km and 80^o^N, acts as a wave guide for the planetary waves to reflect back to the troposphere (Fig. 5d). This can be seen from the curvature in upward component of vertical velocity, between 50^o^–80^o^N. Additionally, the entire high latitude (north of 70^o^N) troposphere (below 20 km) is dominated by the downward component of vertical velocity, which is consistent with reflected and poleward component of the planetary waves (Fig. 2d). During P5, negative wind shear in the mid stratosphere and the meridional curvature in the upward component of vertical velocity weaken significantly (Fig. 5f). However, the downward component of the vertical velocity still remains stronger in the high latitude but in the lower stratosphere (below 15 km).

Therefore, strong negative wind shear around 34 km (5 hPa) act as a reflecting surface for the upward propagating planetary waves at 60^o^W (during P3) to reflect back to the troposphere at 60^o^–120^o^E (during P5). This is evident from the eastward curvature in vertical velocity along the jet axis during P4. Secondly, strong negative wind shear in the middle and lower stratosphere act as a meridional waveguide, which channels the reflected waves back to the troposphere. This can be seen in the meridional curvature in vertical velocity from P3 to P5.

### Effects of Wave Reflection on Tropospheric Weather

**Figure 6:**
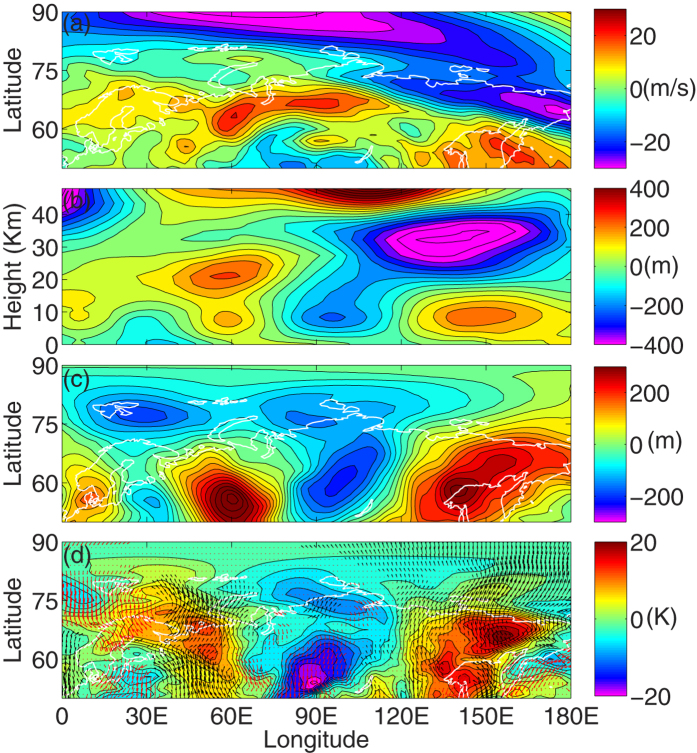
Changes in tropospheric fields between 18^th^ and 15^th^ January 2013. Difference between 18^th^ and 15^th^ January, 2013 for (**a**) Zonal wind at 10 hPa, (**b**) Geopotential heights averaged between 50^o^–80^o^N, (**c**) eddy geopotential height (500 hPa) and (**d**) meridional wind (arrows) and temperature (color contour) at 850 hPa. The black and red arrows in (**d**) represent the southerly and northerly winds, respectively. The maps in the figure are generated using the **MATLAB** software (version: R2012b (8.0.0.783) & URL: www.mathworks.com/products/matlab/?s_tid=srchtitle).

**Figure 7:**
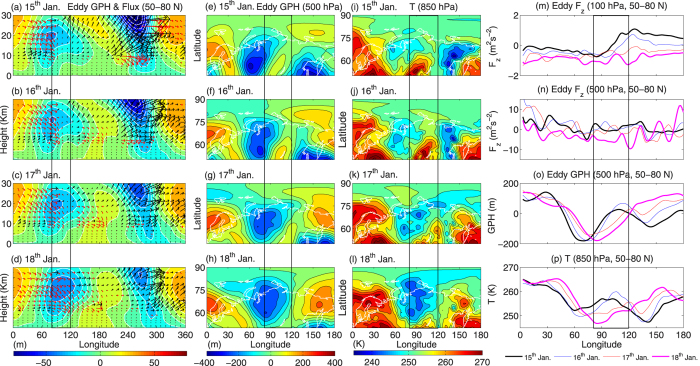
Planetary wave reflection and its impact on tropospheric temperature during 15^th^ to 18^th^ January, 2013. (**a–d**) represent longitude-height eddy geopotential height (color shading) and eddy fluxes (arrows) (mean over 50^o^–80^o^N). The black and red arrows indicate the upward and downward fluxes, respectively. (**e–h**) represent the latitude-longitude eddy geopotential height at 500 hPa. (**I–l**) represent latitude-longitude air temperature at 850 hPa, for 15^th^, 16^th^, 17^th^ and 18^th^ January 2013, respectively. (**m**) Represent eddy F_z_ at 100 hPa, (**n**) represent eddy F_z_ at 500 hPa, (**o**) represent eddy geopotential height at 500 hPa and (**p**) represent air temperature at 850 hPa along the longitude and mean over 50^o^–80^o^N. The black, blue, red and magenta lines in (**m–p**) represent 15^th^, 16^th^, 17^th^ and 18^th^ January, respectively. The longitudinal band between 80^o^ and 120^o^E is marked with black box. The maps in the figure are generated using the **MATLAB** software (version: R2012b (8.0.0.783) & URL: www.mathworks.com/products/matlab/?s_tid=srchtitle).

To illustrate the occurrence of extreme cold event over the central Eurasian continent during mid of January 2013, we have analyzed the differences in background characteristics between the 15^th^ and the 18^th^ of January, i.e., just before and during the peak reflection event, respectively. Differences in different quantities at different altitudes are defined as conditions on January 18^th^ minus conditions on January 15^th^. Figure 6(a) exhibits the differences in zonal wind at 10 hPa. In the high latitude stratosphere, the easterly wind speed increases by 25–30 m/s between 15^th^ and 18^th^ January, 2013 over Eurasia (particularly to the east of 120^o^E) and the background provides favorable conditions for the planetary waves to reflect back to the troposphere. In Fig. 6(b), we have plotted the difference in eddy geopotential height, averaged over 50^o^–80^o^N. On 18^th^, the stationary wave seems propagating downward to the troposphere between 90^o^and 150^o^E, which is indicative from the eastward tilt in the Eurasian trough from 150^o^E in the stratosphere to 90^o^E in the troposphere. This increases the wave activity close to the surface (500–850 hPa). However, negative eddy geopotential height changes do not necessarily means deepening of the trough; it could mean weakening ridge also. Therefore, to confirm the actual changes, daily march of eddy geopotential height from 15^th^ to 18^th^ January is shown in Fig. 7 and we will discuss the individual subplots in the later part of the manuscript.

In the outset, downward components of the Plumb wave activity flux increases the tropospheric (500 hPa) wave activity on 18^th^ January. It is evident from the development of the central Eurasian trough (75^o^–120^o^E) between 50^o^ and 80^o^N on 18^th^ January at 500 hPa (Fig. 6c). The major contribution of the vertical component of the wave activity flux comes from the eddy heat flux term^17^. This near surface increase in wave activity on 18^th^ January, therefore, triggers low level thermal advection adjacent to the surface. Figure 6d illustrates the changes in 850 hPa air temperature and wind over the central Eurasian continent (75^o^–120^o^E) between 18^th^ and 15^th^ January, 2013. Cold polar air was advected southwestward (red arrows) over the central Eurasian continent, and it brought extreme cold weather over Kazakhstan, Southern part of Russia and Northwestern part of China. This region, therefore, experiences a decrease in 20 K temperature within an interval of 72 hours.

To further establish the linkage between planetary wave reflection and anomalous cold weather in the troposphere, we have plotted the daily march of different parameters from 15^th^ to 18^th^ January in Fig. 7. Figure 7a–d exhibit longitude-height section of eddy geopotential height and fluxes (average over 50^o^–80^o^N) for 15^th^, 16^th^, 17^th^ and 18^th^ January, respectively. On 15^th^, the trough around 100^o^E exhibits a complex pattern, with eastward and westward tilt around 50^o^–100^o^E and 100^o^–150^o^E, respectively (Fig. 7a). This is consistent with the downward (red arrows) and upward (black arrows) propagation of the wave activity fluxes in the longitude band mentioned above, respectively. On the other hand, the trough around 300^o^E tilts completely westward and the wave flux seems propagating upward from the troposphere. From 16^th^ to 18^th^ January, the trough between 80^o^E and 120^o^E tilts completely westward and the planetary wave fluxes seems propagating more eastward and downward back to the troposphere (Fig. 7b–d). As a consequence, the reflected component of the planetary wave changes the lower tropospheric (500 hPa) wave activity in the high latitudes (50^o^–80^o^N). Subsequently, the trough in eddy geopotential height between 80^o^E and 120^o^E become stronger on 18^th^ January. On the other hand, around 300^o^E the upward and eastward propagation of the wave fluxes become stronger on 18^th^ January and the trough line tilts completely eastward (Fig. 7b–d). This should be understood as a stratospheric bridge (from 300^o^E to 120^o^E) due to reflection of a zonally propagating wave packet. This is evident from the eastward propagation of the trough and increase in negative geopotential height between 80^o^E and 120^o^E in Fig. 7e–h (shown in black box).

The increase in wave activity near the surface triggers low level thermal advection and brought extreme cold weather on 18^th^ January. Therefore, in the next step we have plotted the latitude-longitude section of near surface (850 hPa) air temperature for 15^th^, 16^th^, 17^th^ and 18^th^ January in Fig. 7i–l, respectively. From 15^th^ to 18^th^ January, in the high latitudes (50^o^–80^o^N), surface air temperature between 80^o^E and 120^o^E (shown in black box) decreases drastically by 20 K within an interval of 72 hours. Furthermore, to quantify the role of downward fluxes due to reflection we have plotted (all for 15^th^, 16^th^, 17^th^ and 18^th^) the eddy F_z_ at 100 hPa, eddy F_z_ at 500 hPa, eddy geopotential height at 500 hPa and air temperature at 850 hPa as a function of longitude (average over 50^o^–80^o^N) in Fig. 7m–p, respectively. On 18^th^ January (magenta line), both the eddy F_z_ at 100 hPa (Fig. 7m) and at 500 hPa (Fig. 7n) exhibit minimum values i.e. stronger downward fluxes due to reflection between 80^o^E and 120^o^E. As a result, lower tropospheric trough (500 hPa) between 80^o^E and 120^o^E strengthens on18^th^ January (magenta line, Fig. 7o). Consequently, air temperature near the surface (850 hPa) and between 80^o^E and 120^o^E decreases at least by ~20 K from its value on 15^th^ January (black line, Fig. 7p).

## Summary and Conclusions

The event of SSW in January-February, 2013 is excited by the combined influence of WN1 and WN2 in the prewarming stage of SSW. In the last 36 years there are only three cases; e.g. 1985/86, 1987/88 and 2012/13 which are due to the combined influence of WN1 and WN2. Moreover, the duration of the warming event is extraordinarily longer (38 days) than its long term climatological mean value. Simultaneously, the stratospheric background during 2012/2013 winter is strongly reflective, provides favorable background for the planetary waves to reflect back to troposphere. All these features make the 2013 event an uncommon one and worth discussing the dynamical evolution of the event. It is evident from Fig. 1a, that a big WN1 pulse on 24^th^ December 2012 in the prewarming stage is likely to precondition the basic state for the WN2 to focus over the polar cap on P2. The stratospheric basic state is very different after the big WN1 pulse and the polar night jet become weaker, thereafter (not shown here).

From Fig. 2, a strong upward propagation of the planetary waves can be noticed on P2, which leads to the reversal of stratospheric polar night jet at 10 hPa, north of 60^o^N, and eventually spread throughout the entire high latitude stratosphere, by the P5. From P3 to P5, the background condition is explicitly favorable for the planetary waves to reflect back to the troposphere and it continues until P6.

The vertical component of the wave activity fluxes at 100 hPa are shown in Fig. 3 and exhibits strong upward propagation of the planetary waves from the ridge over Siberian Tundra and North Pacific on P1, and then from North Atlantic on P2, to constitute a strong WN2 pattern on P3. In the prewarming phase, smaller scale waves, including WN2, being trapped in the troposphere and the WN1 component can only propagate into the stratosphere along the westerly jet^36^. But in the later stage, when the westerly wind is weak enough, the background seems favorable for WN2 to propagate up into the stratosphere and is responsible for the major SSW event on January 2013.

The physical mechanism of planetary wave reflection and the role of negative wind shear during P3, P4 and P5 are explained in Fig. 5. We have plotted the vertical shear of zonal wind to identify the reflecting surfaces around 34 km. An increased eastward curvature in the upward component of vertical wind at 60^o^W (below 34 km) and downward component between 60^o^E and 120^o^E is in agreement with the eastward propagation of upward planetary waves and downward propagation of reflected planetary waves, respectively. Additionally, formation of a meridional waveguide is consistent with an increased meridional curvature in vertical velocity. The poleward curvature in negative wind shear from 32 km and 65^o^N to 20 km and 80^o^N, acts as a wave guide for the planetary waves to reflect back to the troposphere (Fig. 5d). This can be seen from the curvature in upward component of vertical velocity, between 50^o^–80^o^N. During the entire process, planetary waves reflect back over Central Eurasian continent on P4 and this can also be seen from the variation in the tropospheric geopotential field at 500 hPa (Fig. 6c), particularly between 80^o^E and 120^o^E.This near surface increase in wave activity triggers low level thermal advection and the occurrence of extreme cold weather (decrease by ~20 K) around 18^th^ January, 2013 (Fig. 6d). It can be seen that, during the SSW event, increase in wave activity due to reflection seems responsible for the occurrence of cold weather over central Eurasia. Although, the present case study deals with 2013 warming event only, it indicates that the SSWs with stronger WN2 propagation (more barotropic and faster time scale) have the tendency to descend downward. Our results are consistent with Nakagawa and Yamazaki^21^. They have found that, during the development stage of SSW, events that propagate into the troposphere exhibit enhanced upward flux of the WN2, while events that do not propagate downward display reduced WN2 flux. In both events, upward flux of the WN1 is enhanced, but the enhancement is stronger in the non-propagating event. Mitchell *et al*.^22^ showed that for vortex displacement events the largest impact at the surface is observed over the month and anomalous cold temperature (−1.5 K) over North America. For vortex splitting events the mid-stratospheric NAM signal descend down to the surface and the largest effect is observed over northern Eurasia with low temperature anomalies of up to −3 K.

Furthermore, the present analysis provides a hint about increased predictability of the extreme cold events over the central Eurasian continent. The onset of the extreme cold event around 18^th^ January is preceded by a strong WN1 pulse on 24^th^ December (25 days lag) and is followed by a persistent WN2 pulse of constant magnitude from 8^th^ January onwards (10 days lag). On the other hand, the reflective index becomes negative from 4^th^ January onwards (14 days lag), provides a favorable background for WN2 to reflect back to the troposphere. Therefore, not only the WN2 amplitude, the state of high latitude stratosphere (reflective or non-reflective) is essential to make the extreme events more predictable. Moreover, as we discuss in results section and Fig. 5, the region of downward propagation due to reflection is determined by the negative wind shear in the upper stratosphere. In the present case, negative wind shear between 60^o^W and 60^o^E is responsible for the eastward curvature and planetary wave reflection. But if we look Fig. 5a (P3) carefully, strong negative wind shear in the middle-upper stratosphere and vertical velocity provides an impression on the probable regions (180^o^–120^o^W & 0^o^–120^o^E) of reflection and hence the cold wave outbreaks during P4. Therefore, predictability can be improved by monitoring the negative wind shear and strength of vertical velocity, at least few days prior to the main event. However, further works are needed with more number of cases to clarify the condition under which wave reflection has significant impact on the tropospheric extreme weather events.

## Additional Information

**How to cite this article**: Nath, D. *et al*. Dynamics of 2013 Sudden Stratospheric Warming event and its impact on cold weather over Eurasia: Role of planetary wave reflection. *Sci. Rep.* **6**, 24174; doi: 10.1038/srep24174 (2016).
